# Sodium Peroxid Solution

**Published:** 1894-09

**Authors:** 


ARTICLE V.

### SODIUM PEROXID SOLUTION.

The successfull use of sodium peroxid as a bleaching and sterilizing agent depends on the care exercised in making the solution of it. If the solution be made hurriedly by the addition of considerable quantities of the powder to the water at one time, the evolution of heat, due to the energy which attends the combination,produces a rapid elevation of the temperature of the solution. This causes a decomposition of the peroxid, a loss of its loosely combined extra atom of oxigen occurs, and the resulting solution is little more than a solution of sodium hydrate or ordinary caustic soda, which is practically inert as far as bleaching power is concerned. To obviate the rise of temperature and consequent decomposition of the peroxid, the solution must be made slowly, and the vessel in which it is made should be surrounded by ice-water or some cooling mixture; or the solution can be made by gradually dropping the powder on the surface of a piece of ice about the about the size of a chesnut, contained in a small breaker of about one-ounce capacity. Dr. F. T. Van Woert, who has had an extended and satisfactory experience with sodium peroxid as a bleaching and sterilizing agent, kindly furnishes, for the benefit of *Cosmos* readers, the following detailed account of his method of preparing the solution:

"TO THE EDITOR OF THE DENTAL COSMOS.

"*Sir.*—I find, after many experiments, that the most satisfactory solution of sodium peroxid is obtained in the following manner: Take a common tumbler about half full of distilled water, place it in the center of a good-sized pudding-dish, and pour all the cold water around it possible without floating the glass. Add the sodium peroxid in very small portions—-about what could be taken on the point of the large blade of a pocket-knife—dusting it into the water slowly to cause as little agitation as possible and this amount should not be added more than once in a half-hour being careful to have the sodium peroxid finely powdered. This tobe continued till the preparation begins to look opaque as powder is added. Let it stand over night, and it is then ready for use. If a lump about the size of a small bean is dropped into water, you will notice on the margin of the line of agitation a ring of color resembling iodin. A solution made in this way has always proven useless to me. If the peroxid is put in the water as I have suggested, there will be very little surface agitation, and none of the discoloration, the result of which is a solution that has never failed. This takes several days to make, but it will more than pay for the time consumed, in its prompt action as a bleacher and sterilizer. I have placed this solution in the hands of a number of gentlemen, to be used in the treatment of abscessed roots, and to the writing of this not a single failure has been reported. The general impression is that sodium peroxid is for bleaching only, while it is the most valuable preparation ever found for the treatment of dead teeth, if used in the following manner: Cleanse the root-canals of such septic matter as possible to get at with instruments, and dry them with hot air; then carry small ropes of cotton, saturated with a full strength solution, as near the foramen as you can, using orange-wood shaped like fine probes, and cover with a temporary stopping, letting the whole remain for two days, after which wash with hot water, and fill in the usual manner." *F. T Van Woert, in Items of Interest.*

